# Patient-Derived Organoid Models and Precision HIPEC in Diffuse Malignant Peritoneal Mesothelioma: Modeling Heterogeneity to Address Recurrence

**DOI:** 10.3390/ijms27177911

**Published:** 2026-09-04

**Authors:** Eleftherios A. Makris, James K. Ives, Konstantinos Votanopoulos

**Affiliations:** 1 Department of Surgery, Division of Surgical Oncology, Wake Forest University School of Medicine, Winston-Salem, NC 27101, USA; 2 Atrium Health Wake Forest Baptist Comprehensive Cancer Center, Winston-Salem, NC 27157, USA; 3 Wake Forest Organoid Research Center, Winston-Salem, NC 27157, USA

**Keywords:** diffuse malignant peritoneal mesothelioma, cytoreductive surgery, HIPEC, intratumoral heterogeneity, patient-derived organoids, pharmacotyping, precision medicine, tumor microenvironment, chemoresistance, clonal evolution

## Abstract

Diffuse malignant peritoneal mesothelioma (DMPM) is a rare malignancy for which cytoreductive surgery (CRS) with hyperthermic intraperitoneal chemotherapy (HIPEC) is central to treatment in appropriately selected patients. Recurrence remains common even after complete macroscopic cytoreduction. Current HIPEC regimens are protocolized at the institutional and population levels but are not individualized using site-specific molecular or functional tumor biology. We performed a narrative review of clinical, genomic, epigenetic, immune, microenvironmental, and patient-derived organoid evidence relevant to DMPM, CRS/HIPEC, and treatment resistance. Recurrence is multifactorial, with plausible contributions from spatial, histologic, genomic, epigenetic, immune, stromal, and pharmacokinetic heterogeneity. Three primary reports provide direct DMPM organoid evidence, including preliminary demonstrations of patient-specific drug response and discordant responses among anatomically distinct implants. However, these platforms differ biologically, and predictive thresholds, analytical reproducibility, turnaround time, and microenvironmental modeling remain unvalidated. Multi-site organoid pharmacotyping integrated with molecular profiling is therefore a plausible strategy for studying HIPEC resistance. Translation requires a staged pathway encompassing analytical validity, blinded clinical validity, and clinical-utility testing. Precision HIPEC should presently be considered an investigational, validation-ready framework rather than a standard of care.

## 1. Introduction

Diffuse malignant peritoneal mesothelioma (DMPM) is an uncommon and clinically challenging malignancy arising from the mesothelial lining of the peritoneal cavity. Population-level estimates place the annual incidence in the United States in the range of several hundred cases, and presentation is often delayed because early symptoms are nonspecific and may include abdominal distension, ascites, weight loss, and diffuse peritoneal studding [1,2,3]. For patients who are candidates for aggressive locoregional treatment, CRS combined with HIPEC has become a central therapeutic approach in experienced centers, with reported improvements in survival and quality of life compared with historical systemic therapy alone [4,5,6,7,8]. Nevertheless, recurrence remains common even after complete macroscopic cytoreduction. RENAPE (Réseau National de Prise en Charge des Tumeurs Rares du Péritoine) analyses and subsequent recurrence-risk work show that relapse may occur early but can also persist as a long-term risk after initial treatment [9,10]. Repeat CRS/HIPEC may benefit selected patients but is not a feasible salvage option for all patients with recurrent disease [11].

The biological explanation for recurrence after technically adequate CRS/HIPEC remains incompletely defined. Current evidence supports a multifactorial model involving disease burden, completeness of cytoreduction, histology, lymph node status, microscopic residual disease, perfusion heterogeneity, treatment pharmacokinetics, host factors, and tumor-intrinsic biology. DMPM is enriched for tumor-suppressor gene alterations and exhibits meaningful variation in BAP1, NF2, CDKN2A/B, SETD2, LATS2, TP53, and related pathways, with molecular differences between pleural and peritoneal disease [12,13,14,15]. Multi-omic studies of mesothelioma, including the MESOMICS project and transcriptomic analyses containing pleural and peritoneal cohorts, further support the concept that histology alone is an incomplete surrogate for therapeutic biology [16,17]. Immune heterogeneity, stromal heterogeneity, tumor-to-fibroblast plasticity, and dynamic PD-L1 expression add further layers of complexity [18,19,20,21,22].

Patient-derived organoids (PDOs) and patient tumor organoids (PTOs) provide a functional approach to this problem because they can test the integrated response of viable tumor material ex vivo. For consistency in this review, PDO denotes an expandable, predominantly epithelial culture maintained in basement-membrane extract and defined media, whereas PTO denotes a short-term multicellular construct generated directly from dissociated tumor that may retain a broader cellular mixture. Because published terminology varies, platforms are also classified by their cellular composition, expansion capacity, and intended use. Organoid technologies have been applied across multiple cancer types and peritoneal malignancies [23,24,25]. Three primary reports provide direct DMPM evidence: an initial biofabricated short-term tumor-organoid platform, an expandable epithelial organoid system, and a short-term organoid-HIPEC platform [26,27,28]. Pleural mesothelioma organoids derived from tissue or effusions, fibroblast co-cultures, and immune-enhanced systems provide complementary but extrapolative evidence [29,30,31,32,33]. The broader clinical context continues to evolve through molecular pathology, perioperative immunotherapy and circulating tumor DNA analyses, updated ASCO (American Society of Clinical Oncology) guidance for pleural mesothelioma, and a multidisciplinary consensus guideline for peritoneal mesothelioma [34,35,36,37].

This review evaluates whether intratumoral heterogeneity (ITH) may contribute to HIPEC failure in DMPM and whether patient-derived organoid models can convert that biological concept into testable, patient-specific therapeutic hypotheses. Particular attention is given to tumor-suppressor pathway biology, matrix and microenvironmental context, and the molecular effects of hyperthermia because these factors shape both the disease and the proposed assay. Direct DMPM evidence is distinguished from pleural-only and adjacent evidence throughout. The proposed Precision HIPEC framework is an investigational development pathway and not a recommendation for routine clinical implementation outside a prospective protocol.

## 2. Methods and Scope of Review

This narrative review was developed through searches of PubMed, Google Scholar, Scopus/Web of Science, and ClinicalTrials.gov. The search covered records available in each source from database inception through 6 August 2026; no lower date restriction was applied. Search terms included combinations of “diffuse malignant peritoneal mesothelioma,” “peritoneal mesothelioma,” “cytoreductive surgery,” “HIPEC,” “intratumoral heterogeneity,” “patient-derived organoid,” “patient tumor organoid,” “pharmacotyping,” “BAP1,” “NF2,” “CDKN2A,” “MTAP,” “CIMP,” “homologous recombination deficiency,” “single-cell transcriptomics,” “tumor microenvironment,” “PD-L1,” “ctDNA,” “immunotherapy,” and “peritoneal carcinomatosis.” English-language peer-reviewed primary studies, clinical guidelines, translational organoid studies, and large clinical databases were prioritized. Reference lists of key studies and reviews were examined for additional eligible publications. The complete PubMed search strategy is provided in Supplementary Table S1.

Studies were included when they addressed the clinical management or recurrence biology of DMPM, molecular or microenvironmental heterogeneity in mesothelioma, organoid modeling of mesothelioma, or methods directly relevant to hyperthermic pharmacotyping. Case reports were retained only when they supplied mechanistic information unavailable from larger cohorts. Retracted publications were excluded, and reference status was checked using PubMed and publisher records. This was a narrative rather than systematic review; no formal risk-of-bias assessment, meta-analysis, or PRISMA flow diagram was performed.

Because DMPM is rare, the evidence was categorized as: (1) direct DMPM evidence; (2) mixed mesothelioma evidence including peritoneal cases; (3) pleural-only evidence extrapolated to DMPM; or (4) adjacent evidence from other peritoneal malignancies. Direct DMPM organoid evidence currently comprises three primary reports but remains limited by small patient numbers, heterogeneous platforms, and incomplete prospective outcome correlation [26,27,28]. Much of the molecular and single-cell literature derives from pleural disease and is treated as hypothesis-generating when applied to DMPM.

## 3. Clinical Problem: Recurrence After CRS/HIPEC

### 3.1. Recurrence Patterns and the Limits of Clinical Predictors

The recurrence pattern after CRS/HIPEC in DMPM indicates that treatment failure cannot be explained by macroscopic resection status alone. RENAPE data in epithelioid DMPM treated with complete CRS/HIPEC show a substantial early relapse burden and a persistent annual risk of later recurrence [9,10]. A recent 924-patient French multicenter experience further emphasizes the scale and heterogeneity of contemporary DMPM management [38]. Recurrence after CC-0/CC-1 cytoreduction may reflect unrecognized microscopic disease, resistant clonal populations, unfavorable microenvironments, inadequate drug penetration, or clinically occult systemic dissemination.

Established adverse clinical and pathological factors include a high peritoneal cancer index, incomplete cytoreduction, non-epithelioid or biphasic histology, adverse histomorphologic grade, and nodal involvement [5,12,15]. These factors are useful for prognosis and selection, but they do not explain why some apparently favorable patients recur. This residual uncertainty justifies evaluation of tumor-intrinsic and microenvironmental biology while recognizing that ITH is unlikely to be the only mechanism of recurrence.

### 3.2. Standardized Regimens and the Absence of Patient-Level Selection

The continued use of standardized HIPEC protocols is not simply therapeutic inertia. DMPM is rare, randomized regimen-comparison trials are difficult, and institutional practices vary in drug selection, temperature, duration, and carrier solution. Surgical completeness and patient selection strongly influence outcomes, making it difficult to isolate the independent contribution of the perfusion regimen. The 2022 Peritoneal Surface Oncology Group International consensus supports HIPEC after complete CRS and recommends cisplatin plus doxorubicin as the preferred first-line DMPM regimen, while explicitly acknowledging that the underlying level of evidence is low [39].

The translational gap is therefore not an absence of rational or consensus-based protocols; it is the absence of patient-level biological selection. A population-level regimen is applied to spatially distributed disease that may contain multiple histologic, molecular, and functional subclones. A clinically relevant organoid assay must consequently test complete regimens used in practice—including cisplatin–doxorubicin where applicable—under matched temperature, concentration, duration, carrier, and recovery conditions. Evidence from monotherapy comparisons cannot by itself establish superiority among clinical combination regimens. The central question is whether a reproducible ex vivo platform can identify clinically meaningful resistance before HIPEC and, ultimately, whether assay-directed treatment improves patient-centered outcomes.

## 4. Molecular, Spatial, and Temporal Heterogeneity in Mesothelioma

### 4.1. Tumor-Suppressor Biology and Therapeutic Implications

DMPM differs from many solid tumors because recurrent biology is dominated by loss of tumor-suppressor function rather than frequent activating oncogenic drivers. Alterations in BAP1, NF2, CDKN2A/B, SETD2, LATS2, and TP53 recur across mesothelioma datasets, although prevalence and clinical meaning differ between pleural and peritoneal disease [12,34]. The mechanistic consequences of these lesions, rather than their presence alone, generate candidate therapeutic vulnerabilities (Table 1).

BAP1 encodes a nuclear deubiquitinase that regulates chromatin state and participates in DNA-damage responses. BAP1 loss has motivated hypotheses concerning platinum, PARP-inhibitor, and EZH2-directed sensitivity. However, BAP1 loss is not equivalent to canonical homologous-recombination deficiency and is not a validated predictive biomarker for HIPEC or PARP inhibition. Mesothelioma cell-line sensitivity to PARP inhibition was not determined by BAP1 status, and a phase II olaparib study showed limited activity without establishing BAP1 as a determinant of response [40,41]. BAP1-associated hypotheses should therefore be tested as part of integrated functional and molecular models rather than used as stand-alone treatment rules.

NF2 encodes merlin, an upstream regulator of Hippo signaling. Its loss permits YAP/TAZ-TEAD activity and intersects with FAK and mTOR signaling. TEAD-directed approaches remain under early clinical evaluation, whereas the randomized COMMAND study found that maintenance defactinib did not improve progression-free or overall survival in merlin-low pleural mesothelioma [42]. This negative clinical history is important when interpreting pathway-based organoid findings. CDKN2A/B loss may co-delete the adjacent MTAP gene at 9p21, generating a collateral dependency targeted by MTA-cooperative PRMT5 inhibitors such as MRTX1719 [43]. Such molecular features may support trial enrollment or mechanistic stratification but are not established determinants of DMPM HIPEC selection.

### 4.2. MESOMICS and the Limits of Pleural-to-Peritoneal Extrapolation

The MESOMICS project integrated whole-genome sequencing, transcriptomics, and epigenomics in pleural mesothelioma and proposed molecular axes related to ploidy, tumor-cell morphology, adaptive immune response, and CpG island methylator phenotype [16]. This work demonstrates that histologic categories capture only part of the molecular variance. However, MESOMICS is primarily a pleural resource. Its application to DMPM should be framed as a template for future peritoneal multi-omic studies rather than as a validated DMPM classification.

Pleural and peritoneal mesothelioma share tumor-suppressor-driven biology but differ in epidemiology, anatomic growth pattern, treatment context, and immune microenvironment. Dedicated multi-site peritoneal sequencing and transcriptomic datasets are needed before pleural molecular axes can direct peritoneal HIPEC selection.

### 4.3. Spatial and Temporal Heterogeneity

Multi-region sequencing in mesothelioma demonstrates clonal architectures ranging from relatively linear to highly branched [14]. In DMPM, diffuse peritoneal distribution creates an additional sampling problem: implants on the diaphragm, omentum, pelvis, mesentery, and visceral serosa may not be molecularly or functionally equivalent. A single biopsy may therefore underestimate the diversity of residual-risk disease. This supplies a rationale for multi-site sampling, although the number and distribution of samples required to capture clinically meaningful heterogeneity remain unknown.

Temporal heterogeneity is also relevant. Chemotherapy and HIPEC may reshape rather than simply reduce the residual clonal population. Pleural effusion-derived PDOs exposed repeatedly to cisplatin have demonstrated distinct drug-tolerant states, including hypermetabolic adaptation in BAP1-retained models and a hypometabolic, low-proliferation state in BAP1-deficient models [33]. Although these data are pleural and hypothesis-generating for DMPM, they illustrate how treatment can reveal divergent adaptive trajectories. Recurrent DMPM should not be assumed to retain the therapeutic vulnerabilities of pretreatment disease; serial biopsy, ctDNA, and repeat functional testing merit prospective study.

### 4.4. Molecular Determinants of Organoid Drug Response

The molecular variables governing an organoid readout include properties of both the tumor and the assay. Conventional organoids are frequently grown in basement-membrane extract such as Matrigel, whose composition, batch variability, and stiffness influence proliferation, differentiation, drug penetration, and integrin-FAK-YAP/TAZ mechanotransduction [44,45,46,47]. Because NF2/Hippo signaling is central to mesothelioma, matrix-driven YAP activation can confound functional readouts in the tumors for which that pathway is most relevant. Chemically defined, mechanically controlled matrices should therefore be prioritized when cross-site reproducibility is required [45].

Hyperthermia is a distinct molecular stress rather than merely an accelerator of cytotoxicity. Exposure near 42 degrees C activates heat-shock programs, increases membrane permeability and intracellular platinum accumulation, and impairs DNA-repair pathways, including homologous recombination and nucleotide excision repair [48,49,50,51]. Heat-shock responses can also produce thermotolerance, which may blunt repeated exposure. Finally, viability integrates clonally variable transporter, detoxification, and apoptotic biology, including CTR1-mediated platinum uptake; ATP7A/B- and MRP2-mediated efflux; glutathione-dependent detoxification; and BCL-2-family apoptotic thresholds [52,53,54]. Functional response is thus a molecular integrator of genotype, epigenotype, cell state, matrix, and exposure kinetics. That integration is its principal strength, but it also requires rigorous assay standardization.

## 5. Phenotypic, Immune, and Microenvironmental Heterogeneity

### 5.1. Histologic Heterogeneity

Histology remains clinically important in DMPM. Epithelioid disease generally carries a better prognosis than sarcomatoid or biphasic disease, and histomorphologic grading within epithelioid DMPM adds prognostic information [5,15]. Histology is nevertheless an incomplete functional biomarker. Biphasic tumors may contain spatially separated epithelioid and sarcomatoid components, and morphologically similar deposits may differ in drug sensitivity. Organoid-HIPEC findings demonstrating divergent responses among anatomically distinct implants from the same patient offer a functional example of why single-sample histology cannot fully characterize locoregional therapeutic vulnerability [28].

### 5.2. Immune Phenotype and PD-L1 Dynamics

PD-L1 expression and immune contexture are heterogeneous and may change after chemotherapy [18]. A single baseline sample may not represent the immune state of all peritoneal implants, and the post-treatment phenotype may differ from pretreatment disease. These limitations are relevant as immunotherapy is increasingly incorporated into mesothelioma care and perioperative strategies are explored [35,36,37].

Immune heterogeneity should not be reduced to PD-L1. Pleural studies have identified immune subtypes, gradients in tumor-microenvironment composition, and stromal differences [19,20,21]. These data inform model development but cannot substitute for DMPM-specific immune atlases because the peritoneal cavity has a distinct anatomic and immunologic context.

### 5.3. Stromal Plasticity and Co-Culture Requirements

The tumor microenvironment can directly alter drug response. Mesothelioma-associated fibroblasts reduce mesothelioma PDO sensitivity to pemetrexed and cisplatin through IL-6-mediated paracrine signaling [31]. Fibroblast-like cells in mesothelioma may also derive from tumor cells, indicating that part of the stromal compartment can reflect tumor plasticity rather than host reaction alone [22]. These observations support incorporation of matched stromal components when technically feasible.

Pure epithelial organoids may overestimate drug sensitivity in some settings, but existing data are insufficient to invalidate all pure-organoid assays. Stromal and immune co-culture should instead be treated as a prioritized refinement whose incremental predictive value must be tested against simpler models. The added biological fidelity must justify additional time, failure risk, and analytical variability.

## 6. Functional Heterogeneity: Evidence from Patient-Derived Organoid Platforms

### 6.1. Model Taxonomy and Intended Use

The terms PDO and PTO currently encompass biologically different systems. Short-term multicellular PTO constructs are generated directly from dissociated tumors and can preserve a broader cellular mixture while enabling rapid pharmacotyping, but they have limited expansion and longitudinal capacity [26,28]. Expandable epithelial PDOs use basement-membrane extract and defined growth factors to support passaging, genomic analysis, and repeated screening, but culture conditions can select epithelial populations and reduce stromal or immune retention [27]. Effusion-derived and immune- or stroma-enhanced systems occupy additional positions on this spectrum [30,31,32,33].

These model classes should not be treated as interchangeable. Establishment rate, fidelity, turnaround time, passage stability, and response thresholds are platform-specific. Each study and future trial should prespecify the model class, starting cellular composition, matrix, media, passage number, tumor-cell fraction, microenvironmental retention, and intended clinical use. The clinically relevant question is not whether one platform is universally superior, but which level of complexity is analytically reproducible and predictive for a defined decision.

### 6.2. Direct DMPM Organoid Evidence

Three primary reports provide direct DMPM organoid evidence [26,27,28]. Mazzocchi and colleagues biofabricated short-term tumor constructs from two patients (Table 2). One had confirmed epithelioid DMPM, and ex vivo sensitivity to cisplatin plus pemetrexed was concordant with an excellent clinical response. The second was ultimately classified as a well-differentiated papillary mesothelial tumor rather than DMPM and should not be counted as a malignant peritoneal case [26]. This study established early feasibility and patient-level concordance but supplied only one confirmed DMPM observation.

Fang and colleagues established organoids from seven DMPM tumors and one cystic peritoneal mesothelioma, with four lines maintained for more than four passages [27]. The DMPM organoids retained histopathologic and genomic features and demonstrated patient-specific treatment responses. Drug screening was performed across the seven malignant cases, with preliminary retrospective concordance in a subset. These findings support biological fidelity and individualized screening, but they do not constitute blinded or prospective predictive validation.

Forsythe and colleagues evaluated 17 tumors from seven patients using a short-term organoid-HIPEC platform; 16 specimens were evaluable, and results were generated within approximately 10 days [28]. Heated cisplatin met the study’s efficacy criterion in 12 of 16 specimens compared with 7 of 16 for heated mitomycin C. However, pooled post-treatment viability did not differ significantly between the heated cisplatin and heated mitomycin C groups (38.3% versus 50.6%; *p* = 0.10). At the specimen level, heated cisplatin performed significantly better in five specimens and mitomycin C in two. The most important finding is therefore not universal cisplatin superiority but substantial interpatient and intrapatient functional heterogeneity. The study also tested monotherapies rather than the cisplatin–doxorubicin combination favored by current consensus [39].

### 6.3. Pleural Mesothelioma Organoids as Complementary Evidence

Pleural platforms expand the technical evidence base but require cautious extrapolation. Hocking and colleagues generated five long-term cultures from ten malignant pleural effusions obtained from nine patients, supporting minimally invasive and potentially serial assessment [30]. Cioce and colleagues demonstrated that matched fibroblasts can reduce chemotherapy sensitivity through IL-6 signaling [31]. Volpini and colleagues successfully generated 11 PDOs from 15 pleural-fluid or biopsy samples, retained a macrophagic component, and demonstrated heterogeneous responses in PBMC- and pembrolizumab-based testing [32]. These findings support immune-enhanced modeling but do not yet establish a validated DMPM immunotherapy assay.

Useckaite and colleagues used four pleural-effusion PDO models to examine the transition to cisplatin tolerance using transcriptomic and metabolic-flux analyses [33]. The divergent adaptive states observed among PDOs reinforce the value of longitudinal functional models for studying non-genetic resistance. Pleural effusion biology, however, is not interchangeable with peritoneal implant biology, and these results should be used to generate rather than assert DMPM mechanisms.

### 6.4. Technical Limitations Requiring Standardization

Establishment success varies by specimen type, histology, viable tumor fraction, previous therapy, and culture protocol. Growth conditions can select certain clones while losing others. Pure epithelial PDOs may lack stromal and immune compartments, whereas complex co-cultures may decrease throughput and reproducibility. Hyperthermic testing must model clinically relevant regimens, concentration, temperature, exposure time, carrier solution, washout, and recovery intervals. Quality-control criteria should include tumor-cell confirmation, contamination testing, minimum viable biomass, replicate precision, plate controls, and prespecified assay-failure rules.

No validated viability threshold currently defines HIPEC resistance or predicts recurrence. Continuous response metrics should therefore be retained during model development. Any categorical threshold should be derived in a development cohort, locked before evaluation, and validated independently. Cross-platform thresholds should not be assumed to transfer between short-term multicellular constructs and expandable epithelial PDOs.

## 7. Mechanistic Model of HIPEC Failure

**Figure 1:**
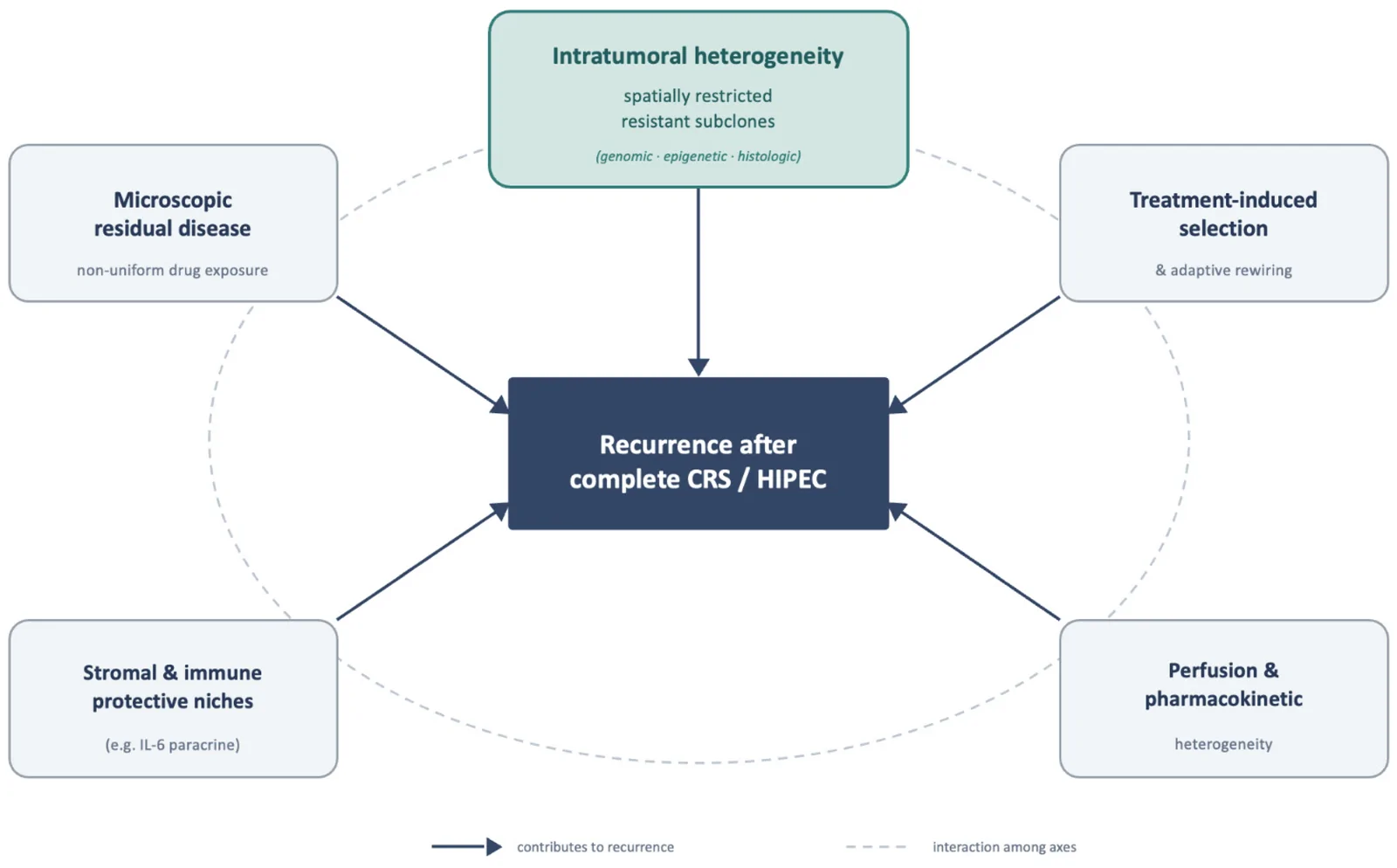
Proposed multifactorial model of CRS/HIPEC failure in DMPM. Intratumoral heterogeneity is one contributor interacting with microscopic residual disease, treatment-induced selection, stromal and immune protection, and perfusion/pharmacokinetic limitations. Solid arrows denote contributions to recurrence, and the dashed ring denotes interactions among axes.

A balanced model of CRS/HIPEC failure should incorporate ITH without treating it as the only explanation for recurrence. Five interacting mechanisms are most plausible (Figure 1). First, microscopic residual disease may remain after CC-0/CC-1 cytoreduction and may not be uniformly exposed to effective drug concentrations. Second, pre-existing resistant subclones may be spatially restricted and missed by single-site sampling. Third, stromal and immune niches may protect tumor cells or modify drug response. Fourth, chemotherapy and hyperthermia may select resistant populations or induce adaptive pathway rewiring. Fifth, pharmacokinetic and perfusion variables may create heterogeneous intraperitoneal exposure.

The organoid contribution is to make components of this model experimentally testable. Multi-site pharmacotyping can quantify whether anatomically distinct implants differ in sensitivity to clinically used regimens. Site-matched genomic, epigenetic, and microenvironmental profiling can then test whether functional resistance aligns with molecular state, clonal architecture, or stromal features. These data can generate a continuous, patient-level resistance index while preserving implant-level heterogeneity for exploratory analyses.

## 8. Proposed Precision HIPEC Framework

### 8.1. Feasibility-Based Multi-Site Sampling

A translational workflow may begin with multi-site tissue acquisition before definitive CRS/HIPEC, most plausibly during a clinically indicated diagnostic laparoscopy or neoadjuvant interval (Table 3). Samples should represent anatomically and macroscopically distinct implants and should be divided for histology, fresh organoid culture, DNA/RNA profiling, and immune or stromal assessment when feasible. Four to six sites may be a reasonable initial feasibility target, but this number is not evidence-based and should not be treated as an optimum. The information gained may depend more on lesion selection than on sample count alone. Pilot protocols should therefore prespecify a diversity-oriented sampling strategy that prioritizes nonredundant lesions from different peritoneal compartments and implants with distinct macroscopic or radiographic features, while ensuring sufficient viable tumor for analysis. Sampling saturation should be evaluated sequentially and in relation to the intended patient-level endpoint. For an aggregate response measure, saturation would be approached when additional strategically selected lesions no longer materially change the patient-level resistance estimate or its uncertainty. For a resistance-detection objective, additional nonredundant lesions should also cease to reveal new discordant or highly resistant phenotypes. Pilot studies should compare lesion-selection strategies to determine which approach captures the greatest functional heterogeneity with the fewest necessary biopsies.

**Table 3:** Translational roadmap for organoid-informed Precision HIPEC.

Development Stage	Principal Objective	Key Measurements	Advancement Criterion
**Feasibility sampling**	Determine whether spatial sampling is practical and informative	Tissue adequacy, anatomic and phenotypic representation, lesion-selection yield, incremental change in the patient-level resistance estimate, detection of new resistant phenotypes, and sampling-related complications	Reproducible acquisition without delaying or compromising standard care
**Analytical validity**	Establish a stable assay under clinically relevant HIPEC conditions	Parent tumor-organoid identity and fidelity, tumor-cell confirmation, success rate, turnaround time, repeatability, inter-laboratory agreement, and quality-control failure	Prespecified identity, fidelity, precision, and reportability criteria achieved
**Blinded clinical validity**	Test whether continuous organoid response predicts outcomes	Adjusted association with RFS; secondary OS and fixed-time recurrence; exploratory lineage-matched recurrence	Prediction model demonstrates reproducible incremental value beyond clinical factors
**Locked independent validation**	Prevent threshold and model optimism	Fixed assay protocol, fixed variables, fixed threshold if required	Performance confirmed in an independent cohort
**Clinical utility**	Determine whether assay-directed regimen selection benefits patients	Randomized comparison of assay-directed versus standard selection; toxicity, delay, RFS, quality of life	Improved patient-centered outcomes without unacceptable delay, toxicity, cost, or inequity

### 8.2. Ex Vivo Regimen Testing and Molecular Integration

Organoids should be exposed to complete, clinically relevant HIPEC regimens under standardized conditions that reproduce concentration, temperature, duration, carrier solution, washout, and recovery. For DMPM, validation panels should include cisplatin–doxorubicin as well as institutionally used alternatives; single-agent testing may be retained for mechanistic decomposition but should not substitute for clinical-regimen testing [39]. Complementary readouts may include viability, apoptosis, DNA damage, and regrowth after treatment.

Molecular profiling should annotate rather than replace pharmacotyping until validated DMPM classifiers exist. A site that is functionally resistant and contains a plausible molecular mechanism may warrant deeper investigation, but neither molecular nor ex vivo findings should change the completeness objective of CRS. Complete cytoreduction remains the operative goal for every eligible patient. In early studies, resistant-site results should support enhanced sampling, mechanistic profiling, surveillance hypotheses, or trial eligibility—not selective undertreatment or unvalidated changes to resection.

### 8.3. Staged Validation and Decision Logic

**Figure 2:**
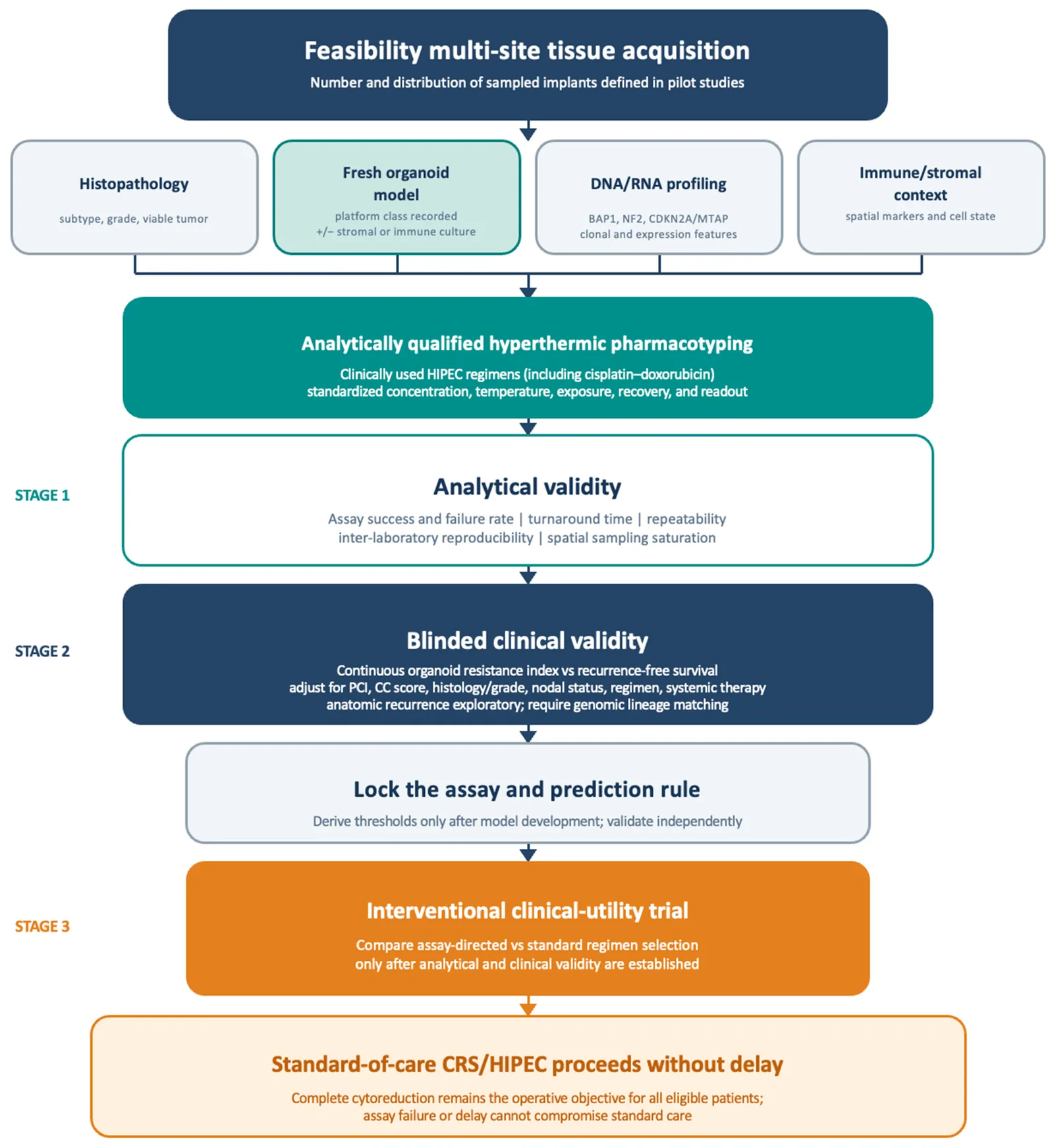
Proposed staged validation pathway for organoid-informed Precision HIPEC. Feasibility-based multi-site sampling is followed by parent tumor-organoid identity and fidelity quality control, including sample-identity and contamination controls, histopathologic or lineage confirmation, and assessment of retention of defining molecular features. Only models satisfying prespecified QC criteria proceed to clinically relevant hyperthermic pharmacotyping and analytical qualification. Blinded clinical-validity testing uses a continuous resistance index and adjusted recurrence-free survival analysis. A prediction rule is locked before independent validation and any subsequent interventional clinical-utility trial. Standard-of-care CRS/HIPEC proceeds without delay throughout development.

Before organoid-guided HIPEC can be used in routine practice, future studies must establish three separate requirements: analytical reproducibility, clinical validity through blinded correlation of prespecified organoid responses with patient outcomes after the corresponding regimen, and clinical utility through evidence that assay-directed regimen selection improves outcomes. Precision HIPEC should therefore be developed through three sequential gates (Figure 2). Stage 1 establishes analytical validity: assay success and failure rates, turnaround time, repeatability, inter-laboratory reproducibility, effects of specimen handling, and spatial sampling saturation. Stage 2 establishes clinical validity in a blinded, non-directing observational cohort. The primary analysis should relate a continuous organoid resistance index to recurrence-free survival while adjusting for peritoneal cancer index, completeness of cytoreduction, histology and grade, nodal status, HIPEC regimen, and systemic therapy. Overall survival, recurrence at fixed time points, and assay feasibility are important secondary outcomes.

The anatomic site of first recurrence is a fragile primary endpoint because sampled lesions are removed and recurrence geography reflects surgical, perfusion, reseeding, and surveillance factors. Site-resolved recurrence should therefore be exploratory and interpreted as clonal continuity only when genomic or spatial lineage matching links recurrent disease to a baseline deposit. After a response model is developed, its variables and any categorical threshold should be locked and independently validated. Stage 3 is a prospective clinical-utility trial comparing assay-directed with standard regimen selection. Standard care must proceed without delay whenever the assay fails, is late, or is non-informative. Analytical reproducibility or outcome correlation alone would not justify assay-directed treatment.

### 8.4. Implementation Barriers and a Realistic Operational Scenario

Time is the most immediate operational constraint. Turnaround is platform-dependent: short-term multicellular constructs have generated DMPM pharmacotyping results within approximately 10 days, whereas expandable cultures may require several weeks [28]. A clinically actionable result is therefore most plausible within an existing neoadjuvant interval or following clinically indicated staged laparoscopy, rather than during a single-stage CRS/HIPEC procedure. Additional sampling must be justified by study value and cannot expose patients to an otherwise unnecessary operation without a favorable research risk–benefit assessment.

Establishment failure is a second constraint. Success depends on viable tumor content, previous treatment, tissue handling, and platform. The workflow must therefore ensure that standard treatment is never contingent on a successful assay. Initial validation is most realistic at high-volume centers with embedded organoid laboratories and coordinated pathology, surgery, and biobanking workflows. Multi-institutional development will require harmonized collection kits, temperature-controlled logistics, assay controls, reporting timelines, data standards, regulatory oversight, cost analysis, and equitable access.

## 9. Clinical Trials and Biomarker Development

Early validation should be observational and blinded: all patients receive protocol-defined standard care, and organoid results remain non-directing. Prespecified feasibility outcomes should include the proportion of patients with a reportable assay, time to result, reasons for failure, replicate precision, and cross-site reproducibility. Clinical-validity analyses should prioritize continuous functional response rather than an arbitrary early dichotomy. Sample-size calculations must account for DMPM rarity, recurrence rate, assay failure, center effects, competing risks, and covariate adjustment.

Candidate annotations include BAP1 and NF2 status, CDKN2A/B and MTAP loss, CIMP-related profiles, spatial PD-L1 expression, immune subtype, stromal IL-6 signaling, ctDNA minimal residual disease, and organoid-derived response features. Functional response may ultimately outperform a single molecular alteration because it integrates genomic, epigenetic, cell-state, and microenvironmental effects. However, molecular and functional variables must be prespecified, and model optimism must be controlled through internal resampling followed by independent validation.

Serial monitoring should be incorporated when feasible. ctDNA may detect molecular residual disease or clonal recurrence earlier than imaging, whereas repeat biopsy and organoid testing at recurrence could identify acquired resistance [35]. Longitudinal sampling is particularly relevant if systemic therapy or HIPEC changes clonal composition. As with baseline testing, serial organoid analysis should not guide salvage treatment outside a trial until analytical and clinical validity are established.

## 10. Limitations of Current Evidence

Direct DMPM organoid evidence is promising but comprises three small primary reports with heterogeneous biological platforms [26,27,28]. The earliest report contributes only one confirmed DMPM case, and later studies provide preliminary rather than prospective outcome correlation. Much of the molecular, single-cell, and immune-organoid literature comes from pleural mesothelioma and cannot be treated as peritoneal-specific evidence. Culture conditions, matrix, passage number, assay timing, cellular composition, and readout vary across platforms. Expansion may favor viable, proliferative, or epithelial populations and underrepresent aggressive, resistant subclones. Pure epithelial PDOs omit stromal, immune, vascular, and pharmacokinetic determinants, whereas complex co-cultures may sacrifice throughput and reproducibility.

The proposed framework also has clinical limitations. Multi-site sampling can add procedural risk, cost, and logistical burden. Neoadjuvant therapy during assay development may change the biology being measured. Ex vivo exposure cannot fully reproduce intraperitoneal distribution, tissue penetration, host metabolism, or surgical factors. There is no validated response threshold, and DMPM rarity makes independent multi-institutional validation difficult. Recurrence remains multifactorial even if tumor biology is characterized accurately. These limitations support a staged validation program rather than immediate clinical adoption.

## 11. Conclusions

Recurrence after CRS/HIPEC remains a major problem in DMPM. Spatial, molecular, immune, stromal, functional, and pharmacokinetic heterogeneity plausibly contribute to treatment failure, but current evidence does not establish ITH as the dominant mechanism in every patient. Patient-derived organoid models provide a way to measure functional heterogeneity and investigate clinically relevant HIPEC regimens under controlled conditions. Their most compelling near-term uses are mechanistic discovery, analytical platform development, and blinded correlation of continuous response with recurrence outcomes.

A Precision HIPEC paradigm integrating feasibility-based multi-site sampling, organoid pharmacotyping, molecular annotation, microenvironmental modeling, and longitudinal surveillance is scientifically plausible. Its clinical value will depend on analytical validity, independent clinical validation, and randomized evidence that assay-directed regimen selection improves outcomes without unacceptable delay, toxicity, cost, or inequity. Until those conditions are met, organoid-informed HIPEC should remain a high-priority investigational framework rather than an established standard of care.

## Figures and Tables

**Table 1:** Selected molecular and functional features relevant to precision-treatment hypotheses in DMPM.

Feature	Main Relevance to Precision HIPEC	Evidence Level	Key Limitation
**BAP1 alteration or loss**	Candidate DNA damage and epigenetic vulnerability	Frequent in DMPM; predictive evidence is inconsistent	Not equivalent to canonical HR deficiency and not a stand-alone treatment selector
**NF2/Hippo alteration**	YAP/TAZ-TEAD and FAK pathway hypotheses	Mechanistic rationale; emerging TEAD strategies; negative randomized FAK history	Pathway redundancy and adaptive resistance
**CDKN2A/B and MTAP loss**	Trial eligibility for MTAP-loss synthetic-lethal strategies	Biologically rational; not validated for DMPM HIPEC selection	Investigational use only
**CIMP and related molecular axes**	Potentially distinct drug-response states	Evidence derives mainly from pleural multi-omics	DMPM-specific spatial validation is needed
**PD-L1 and immune contexture**	Immune modeling and treatment-sequencing hypotheses	Dynamic and heterogeneous; limited DMPM-specific data	A single sample may misclassify the immune phenotype
**ECM composition and stiffness**	Drug penetration and integrin-FAK-YAP/TAZ signaling	Established in organoid biology; incompletely standardized in mesothelioma	Matrix properties must be controlled across sites and batches
**Hyperthermic stress**	Heat-shock signaling, DNA repair, platinum uptake, and thermotolerance	Mechanistic in vitro evidence	Clinical exposure and recovery conditions must be reproduced
**Functional organoid response**	Integrates multiple tumor and microenvironmental response determinants	Promising; three small direct DMPM reports	No locked clinical threshold or prospective utility evidence

**Table 2:** Mesothelioma organoid studies and translational lessons for Precision HIPEC.

Study	Model and Evidence Level	Main Finding	Key Limitation
**Mazzocchi et al.** [26]	Short-term multicellular platform; direct DMPM evidence (one confirmed case)	Feasibility and patient-level drug-response concordance	Single confirmed DMPM case; no prospective validation
**Fang et al.** [27]	Expandable epithelial PDOs; direct DMPM evidence (seven malignant cases)	Parent-tumor fidelity and heterogeneous drug responses	Small cohort; culture selection; limited retrospective concordance
**Forsythe et al.** [28]	Short-term PTO-HIPEC platform; direct DMPM evidence (16 specimens from seven patients)	Rapid results and marked interpatient and intrapatient response heterogeneity	Monotherapy comparison; pooled cisplatin-versus-mitomycin difference not significant; no threshold validation
**Hocking et al.** [30]	Effusion-derived expandable PDOs; pleural extrapolative evidence	Feasibility of minimally invasive and serial modeling	Peritoneal applicability unknown; modest establishment rate
**Cioce et al.** [31]	PDO-fibroblast co-culture; pleural extrapolative evidence	IL-6-mediated stromal reduction in chemotherapy sensitivity	Added complexity; no validated rapid workflow
**Volpini et al.** [32]	Microenvironment-retaining PDOs; pleural extrapolative evidence	Macrophage retention and heterogeneous immune responses	Immune assay not validated for treatment selection
**Useckaite et al.** [33]	Longitudinal cisplatin-tolerance PDOs; pleural mechanistic evidence	Distinct metabolic and transcriptomic tolerance states	Small mechanistic cohort; not clinically predictive

## Data Availability

No new data were created or analyzed in this study. Data sharing is not applicable to this article.

## Supplementary Materials

The following supporting information can be downloaded at: https://www.mdpi.com/article/10.3390/ijms27177911/s1.
