# Supplementary material for: Patient-Derived Organoid Models and Precision HIPEC in Diffuse Malignant Peritoneal Mesothelioma: Modeling Heterogeneity to Address Recurrence
Source: Int J Mol Sci. 2026 Sep 4;27(17):7911. doi: 10.3390/ijms27177911 (PMC13566438; doi:10.3390/ijms27177911)
Supplement: Supplementary file 1 [file ijms-27-07911-s001.zip › ijms-4516724-supplementary.pdf]

Supplementary Materials

Table S1. Complete PubMed search strategy

Database/platform: PubMed (National Library of Medicine). Search date: 6 August 2026. Coverage: database inception through 6 August 2026; no lower date restriction. Search-stage filters: no language or study-design filters were applied. During screening, English-language peer-reviewed primary studies, clinical guidelines, translational organoid studies, and large clinical databases were prioritized. The searches were run as separate concept blocks and screened together; reference lists of key studies and reviews were examined for additional eligible reports.

**Search domain**	**Exact PubMed search string**
**1. DMPM clinical management, recurrence, CRS, and HIPEC**	(("Mesothelioma, Malignant"[Mesh] OR mesothelioma*[Title/Abstract]) AND ("Peritoneal Neoplasms"[Mesh] OR peritoneal[Title/Abstract])) AND ("Cytoreduction Surgical Procedures"[Mesh] OR cytoreductive surg*[Title/Abstract] OR CRS[Title/Abstract] OR "Hyperthermic Intraperitoneal Chemotherapy"[Mesh] OR HIPEC[Title/Abstract] OR recurrence[Title/Abstract] OR outcome*[Title/Abstract])
**2. Molecular, spatial, immune, and microenvironmental heterogeneity**	("Mesothelioma, Malignant"[Mesh] OR mesothelioma*[Title/Abstract]) AND (heterogene*[Title/Abstract] OR BAP1[Title/Abstract] OR NF2[Title/Abstract] OR CDKN2A[Title/Abstract] OR MTAP[Title/Abstract] OR CIMP[Title/Abstract] OR "homologous recombination"[Title/Abstract] OR "single-cell"[Title/Abstract] OR transcriptom*[Title/Abstract] OR "tumor microenvironment"[Title/Abstract] OR "tumour microenvironment"[Title/Abstract] OR PD-L1[Title/Abstract] OR ctDNA[Title/Abstract] OR immunotherap*[Title/Abstract])
**3. Mesothelioma organoids and functional pharmacotyping**	("Mesothelioma, Malignant"[Mesh] OR mesothelioma*[Title/Abstract]) AND

	("Organoids"[Mesh] OR organoid*[Title/Abstract] OR pharmacotyp*[Title/Abstract] OR "ex vivo"[Title/Abstract] OR hypertherm*[Title/Abstract])
**4. Adjacent peritoneal-malignancy organoid evidence**	("Peritoneal Neoplasms"[Mesh] OR (peritoneal[Title/Abstract] AND metastas*[Title/Abstract]) OR "peritoneal carcinomatosis"[Title/Abstract]) AND ("Organoids"[Mesh] OR organoid*[Title/Abstract] OR pharmacotyp*[Title/Abstract]) AND (HIPEC[Title/Abstract] OR hypertherm*[Title/Abstract] OR intraperitoneal[Title/Abstract])

Abbreviations: CRS, cytoreductive surgery; DMPM, diffuse malignant peritoneal mesothelioma; HIPEC, hyperthermic intraperitoneal chemotherapy.
